# Antibodies to Heteromeric Glycolipid Complexes in Guillain-Barré Syndrome

**DOI:** 10.1371/journal.pone.0082337

**Published:** 2013-12-16

**Authors:** Simon Rinaldi, Kathryn M. Brennan, Gabriela Kalna, Christa Walgaard, Pieter van Doorn, Bart C. Jacobs, Robert K. Yu, Jan-Eric Mansson, Carl S. Goodyear, Hugh J. Willison

**Affiliations:** 1 Institute of Infection, Immunity and Inflammation, University of Glasgow, Glasgow, United Kingdom; 2 Bioinformatics, Beatson Institute for Cancer Research, Glasgow, United Kingdom; 3 Department of Neurology, Erasmus Medical Centre, University Medical Center, Rotterdam, The Netherlands; 4 Department of Immunology, Erasmus Medical Centre, University Medical Center, Rotterdam, The Netherlands; 5 Institute of Molecular Medicine and Genetics, Medical College of Georgia, Georgia Health Sciences University, Augusta, Georgia, United States of America; 6 Laboratory Medicine/Clinical Chemistry, Sahlgren's University Hospital, Molndal, Sweden; Innsbruck Medical University, Austria

## Abstract

Autoantibodies are infrequently detected in the sera of patients with the demyelinating form of Guillain-Barré syndrome most commonly encountered in the Western world, despite abundant circumstantial evidence suggesting their existence. We hypothesised that antibody specificities reliant on the *cis* interactions of neighbouring membrane glycolipids could explain this discrepancy, and would not have been detected by traditional serological assays using highly purified preparations of single gangliosides. To assess the frequency of glycolipid complex antibodies in a Western European cohort of patients GBS we used a newly developed combinatorial glycoarray methodology to screen against large range of antigens (11 gangliosides, 8 other single glycolipids and 162 heterodimeric glycolipid complexes). Serum samples of 181 patients from a geographically defined, Western European cohort of GBS cases were analysed, along with 161 control sera. Serum IgG binding to single gangliosides was observed in 80.0% of axonal GBS cases, but in only 11.8% of cases with demyelinating electrophysiology. The inclusion of glycolipid complexes increased the positivity rate in demyelinating disease to 62.4%. There were 40 antigens with statistically significantly increased binding intensities in GBS as compared to healthy control sera. Of these, 7 complex antigens and 1 single ganglioside also produced statistically significantly increased binding intensities in GBS versus neurological disease controls. The detection of antibodies against specific complexes was associated with particular clinical features including disease severity, requirement for mechanical ventilation, and axonal electrophysiology. This study demonstrates that while antibodies against single gangliosides are often found in cases with axonal-type electrophysiology, antibodies against glycolipid complexes predominate in cases with demyelinating electrophysiology, providing a more robust serum biomarker than has ever been previously available for such cases. This work confirms the activation of the humoral immune system in the dysimmune disease process in GBS, and correlates patterns of antigen recognition with different clinical features.

## Introduction

Current evidence suggests that Guillain-Barré syndrome (GBS) is caused in some cases by autoantibodies arising via microbial molecular mimicry [1–4]. Certain antibodies clearly correlate with particular disease subtypes [5] [6]; however, these clinical-serological relationships are not absolute. GBS cohorts dominated by demyelinating/AIDP-type electrophysiology have no prevalent antibody association, and no serum biomarker is available to reliably support diagnosis [7,8]. Furthermore, there are inconsistencies between the ganglioside antigen tissue distribution and disease phenotype [9], leading some to debate the pathological significance of detected antibodies [10,11]. 

In addition to serological reactions with single ganglioside or glycolipid species, it has recently been observed that certain GBS-associated autoantibodies may only bind to ganglioside complexes (GSC). GSC antibodies react with mixtures of two different gangliosides, whilst failing to recognise either component ganglioside alone, [12,13]. This concept builds on the long standing hypotheses of a lectin-binding “clustered saccharide patch” [14]. Following on from these serological studies, the pathological importance of GM1-complex antibodies [15,16] and the modulatory effects of GSCs on other lectin-carbohydrate interactions [17,18] have both been demonstrated. Nevertheless, the precise physico-chemical nature of GSCs, and how they influence lectin and antibody binding, still remains unknown. Although biophysical studies show that *cis* (i.e. side-to-side) interactions between neighbouring glycolipids in artificial and living membranes do occur [19,20], the term ‘glycolipid complex’ is here used simply to denote the mixture of two different glycolipids applied to a membrane.

The relevance of GSC antibodies to Western GBS cohorts dominated by demyelinating pattern electrophysiology, as compared to Japanese cohorts with a greater proportion of axonal GBS, is unexplored. Since screening serum samples for anti-GSC reactivity is confounded by technical constraints, we developed a combinatorial glycoarray whereby very small quantities of glycolipid are sprayed onto polyvinylidene difluoride (PVDF) membranes and then probed with serum [17], allowing us to screen a large cohort of sera from GBS patients against a large number of single and complex glycolipid antigens. This approach enabled us to fulfil our aim of delineating the glycolipid complex antibody profile in a geographically defined cohort of Western European GBS patients, providing a more robust serum biomarker than has ever been previously available, and confirming the activation of the humoral immune system in the disease.

## Methods

### Patient cohort

#### Patients and serum samples

225 GBS patients were recruited from centres across the Netherlands, Belgium and Germany between 1994 and 2000 for a clinical trial [21]. Inclusion criteria were fulfilment of the NINDS diagnostic criteria for GBS [7], GBS disability score of 3 or more (unable to walk 10m unaided) [22], and onset of weakness within two weeks before randomization. Ethical approval was obtained from each centre in the Netherlands, Belgium and Germany which participated in the original clinical trial, and informed written consent was obtained from each individual patient. GBS serum samples used were obtained within four weeks from onset of weakness and before start of treatment. Only sera from this one time point were available for analysis. For the current study sufficient amounts of pre-treatment serum had to be available for analysis. Serum samples from 74 healthy controls (hospital and laboratory workers with no relevant past medical history) and 87 patients with other neurological diseases (“OND controls”, 40 with multiple sclerosis (MS), and 47 other ‘non-inflammatory’ neurological diseases – ONND, collected as part of a previous study [23], see table S1 for details) were also obtained. Sera were stored at -80°C and defrosted shortly before each use. 

#### Data collection

Baseline characteristics and clinical features were collected prospectively. Electrophysiological examination was performed at one time point, and disease classification based on accepted current criteria [24]. Serological screening had been previously performed for GM1, GD1a and GQ1b ganglioside antibodies using a conventional ELISA technique. By this assay a positive serum is defined by an optical density for a ganglioside coated well more than 0.2 greater than uncoated control well, at a titre of 1:100 or greater [25]. 

### Combinatorial glycoarray

A TLC autosampler was used to apply glycolipid onto PVDF membranes as previously described [26]. Glycolipids and their sources, and the resulting GSC antigens created, are listed in Table **1**. LM1 and sulfated glucuronyl paragloboside (SGPG) were extracted and purified as described [27,28]. Complexes (1:1 by weight) were created by admixing equal volumes of the component glycolipids. Arrays were blocked and then incubated with sera diluted 1 in 100. After washing, rabbit anti-human IgG horseradish peroxidase conjugated secondary antibody (Dako) was applied at 1:30,000 dilution. The IgG response alone was assessed as extensive previous observations have indicated that IgG is the critical immunoglobulin class in GBS. Binding was detected by enhanced chemiluminescence (ECL+, Amersham/GE Healthcare). Radiographs were digitised by flatbed scanning and spot intensity calculated using TotalLab image analysis software (Nonlinear Dynamics). The order of analysis of the sera was determined by random number generation, the sera coded, and the investigator performing image analysis blinded to the identity of the serum applied to each membrane.

**Table 1:** Antigens included in the glycoarray.

1	SM*	46	*PS:GM1+*	91	SGPG:GD3	136	GA1:Sulfatide+
2	PS*+	47	PS:GD1a	92	SGPG:GQ1b	137	*Sulfatide :GalC*
3	Globoside***	48	*PS:GD1b+*	93	SGPG:GT1b	138	*Sulfatide :GM1+*
4	CTH***	49	PS:GD3	94	**SGPG:Sulfatide+**	139	Sulfatide :LM1
5	**SGPG+**	50	PS:GQ1b	95	GM2:GM3	140	Sulfatide :GD3
6	GM2*	51	PS:GT1b	96	GM2:GD2	141	Sulfatide :GD1a
7	GM3*	52	**PS:Sulfatide+**	97	GM2:GA1	142	*Sulfatide :GD1b+*
8	GD2*	53	Globoside:CTH	98	GM2:GalC	143	**Sulfatide :GT1a**
9	GA1*	54	Globoside:SGPG	99	GM2:LM1	144	Sulfatide :GT1b
10	GalC***	55	Globoside:GM2	100	GM2:GM1	145	Sulfatide :GQ1b
11	LM1	56	Globoside:GM3	101	GM2:GD1a	146	*GalC:GM1+*
12	GM1*+	57	Globoside:GD2	102	GM2:GD1b	147	GalC:LM1
13	GD1a*	58	Globoside:GA1	103	GM2:GD3	148	GalC:GD3
14	GD1b*+	59	Globoside:GalC	104	GM2:GQ1b	149	GalC:GD1a
15	GD3*	60	Globoside:LM1	105	GM2:GT1b	150	GalC:GD1b
16	GQ1b**	61	Globoside:GM1	106	GM2:Sulfatide	151	*GalC:GT1a*
17	GT1b*	62	Globoside:GD1a	107	GM3:GD2	152	GalC:GT1b
18	Sulfatide*	63	Globoside:GD1b	108	GM3:GA1	153	GalC:GQ1b
19	GT1a***	64	Globoside:GD3	109	GM3:GalC	154	*GM1:LM1+*
20	SM:PS	65	Globoside:GQ1b	110	GM3:LM1	155	*GM1:GD3+*
21	SM:Globoside	66	Globoside:GT1b	111	*GM3:GM1+*	156	*GM1:GD1a+*
22	SM:CTH	67	Globoside:Sulfatide	112	GM3:GD1a	157	*GM1:GD1b+*
23	SM:SGPG	68	CTH :SGPG	113	*GM3:GD1b+*	158	*GM1:GT1a*
24	SM:GM2	69	CTH :GM2	114	GM3:GD3	159	GM1:GT1b
25	SM:GM3	70	CTH :GM3	115	GM3:GQ1b	160	GM1:GQ1b
26	SM:GD2	71	CTH :GD2	116	GM3:GT1b	161	LM1:GD3
27	SM:GA1	72	CTH :GA1	117	GM3:Sulfatide	162	LM1:GD1a
28	SM:GalC	73	CTH :GalC	118	*GD2:GA1+*	163	*LM1:GD1b+*
29	SM:LM1	74	CTH :LM1	119	GD2:GalC	164	*LM1:GT1a*
30	*SM:GM1*	75	CTH :GM1	120	GD2:LM1	165	LM1:GT1b
31	SM:GD1a	76	CTH :GD1a	121	GD2:GM1	166	LM1:GQ1b
32	SM:GD1b	77	CTH :GD1b	122	GD2:GD1a	167	GD3:GD1a
33	SM:GD3	78	CTH :GD3	123	GD2:GD1b	168	GD3:GD1b
34	SM:GQ1b	79	CTH :GQ1b	124	GD2:GD3	169	GD3:GT1a
35	SM:GT1b	80	CTH :GT1b	125	GD2:GQ1b	170	GD3:GT1b
36	**SM:Sulfatide+**	81	**CTH :Sulfatide+**	126	GD2:GT1b	171	GD3:GQ1b
37	PS:Globoside	82	SGPG:GM2	127	*GD2:Sulfatide+*	172	GD1a:GD1b
38	PS:CTH	83	SGPG:GM3	128	GA1:GalC	173	GD1a:GT1a
39	**PS:SGPG+**	84	SGPG:GD2	129	*GA1:LM1+*	174	GD1a:GT1b
40	PS:GM2	85	SGPG:GA1	130	*GA1:GM1+*	175	GD1a:GQ1b
41	PS:GM3	86	*SGPG:GalC*	131	*GA1:GD1a+*	176	GD1b:GT1a
42	PS:GD2	87	SGPG:LM1	132	GA1:GD1b	177	GD1b:GT1b
43	*PS:GA1+*	88	*SGPG:GM1+*	133	*GA1:GD3+*	178	GD1b:GQ1b
44	*PS:GalC*	89	SGPG:GD1a	134	GA1:GQ1b	179	GT1a:GT1b
45	PS:LM1	90	*SGPG:GD1b+*	135	GA1:GT1b	180	GT1a:GQ1b
						181	GT1b:GQ1b

19 single glycolipids (numbers 1 to 19) and 172 1:1 complexes (20 to 181) were included in the assay in two separate screens. First an 11x11 array was performed, comprising antigens numbered 10 to 19and the 45 1:1 heterodimeric complexes formed by these glycolipids. The remaining antigens were assessed on a 28x10 array. For each spot 0.1µl per glycolipid (at 100µg/ml in methanol) was applied. Lipids were obtained from *Sigma, Poole, UK, **Accurate Chemical &Scientific, Westbury, USA, and ***Matreya, Pleasant Gap, USA. LM1 and SGPG were prepared and purified in house. +antigens with significantly increased binding *frequency* with GBS versus OND control sera. Emboldened antigens had were significantly associated with GBS versus healthy controls following Bonferroni correction, italicised antigens were significant only following less stringent Benjamini and Hochberg step-up correction for multiple comparisons.. (SM – sphingomyelin, PS – phosphatidylserine, GalC – galactocerebroside, GA1 – asialo-GM1, sul – sulfatide, CTH – trihexosylceramide (ceramide trihexosides), SGPG - sulfated glucuronyl paragloboside)

For practical purposes clinical samples were screened in batches. Initially, GBS cases and healthy controls were screened using an 11x11 and then 28x10 array (Table **1**). Antibody-antigen interactions statistically significantly associated with GBS, in comparison with healthy controls, were then re-evaluated with the inclusion of all 87 disease control sera.

### Statistical analysis

Initial analysis was performed using the Chi-squared test on binary data (antigen binding present or absent) to investigate overall antibody-antigen-disease associations. Intensity values were used to produce heatmaps, which underwent iterative clustering to reveal patterns of binding. Differences in binding intensity between antigen groups (single gangliosides, glycolipids and glycolipid complexes) were assessed by Kruskal-Wallis with post-hoc analysis using the Mann-Whitney test. To compare the binding intensities achieved by GBS and control sera to the multiple different individual antigens on the array, raw intensity values were first logarithmically transformed. A Mixed Model Analysis of Variance (ANOVA) was then used to make multiple comparisons (in which zero results were considered as the lowest value for a continuum of binding intensities). This approach was also used to assess for associations between clinical features and antigen binding intensity, and allows for the fact that intensity values of some antigens are likely to be inter-related. The problems associated with analysing data with multiple zero values were addressed by considering and comparing results obtained by the Chi-squared and ANOVA and Mann-Whitney approaches. The latter two methods were used after replacing zero intensity values with a small positive value. Both Bonferroni and Benjamini and Hochberg step-up correction techniques were used to correct for multiple comparisons. The former is the most conservative correction for multiple comparisons, but therefore comes at the cost of a higher risk of false negatives. As such, the less stringent and Benjamini and Hochberg step-up technique was also used in certain exploratory situations. 

## Results

### Patient cohort

Sufficient amounts of serum were available from 181 of 225 patients. Clinical data were available for 180 of these 181 patients. 103 were male (57.2%) and 77 female (42.8%). The mean age of patients was 52.6 years (range 7 to 89). The mean/median age was 44.2/44 for healthy controls, 55.7/56 for OND controls, and 50.4/49 (range 25-83) for controls overall. Electrophysiological data were available for 172 out of 181 patients. A single set of nerve conduction studies (NCS) was performed at a median of 17 days (inter-quartile range 11-21 days) following the onset of weakness. According to the Hadden criteria [24], the majority were classified either as AIDP (85 cases, 49.4%) or equivocal (73 cases, 42.4%). Equivocal patients had a definite clinical diagnosis of GBS, but in these instances NCS were unable to distinguish demyelinating or axonal subtypes following the single timepoint testing that characterised the study design. Only 5 patients had electrophysiology indicative of AMAN (Acute Motor Axonal Neuropathy, 5.6% of characterised cases). Four cases had normal neurophysiology and in 5 the nerves were unexcitable. There were 5 deaths (2.8%) and 41 patients (22.8%) required mechanical ventilation at some point. 

### Glycoarray Assay Performance

The inter- and intra-assay coefficients of variation (CV) for a 10x10 glycoarray had been previously calculated at 4.1% and 8.6% respectively, using an anti-GM1 monoclonal antibody [17]. One positive GBS serum identified in the first screen of the 28x10 assay was retested on 12 occasions over a 3 month time period during the assessment of GBS and healthy control samples. The inter-assay CV for this sample over this period, involving multiple freeze-thaw cycles and different batches of reagents, was 13.4%.

### Initial Screening

**Figure 1:**
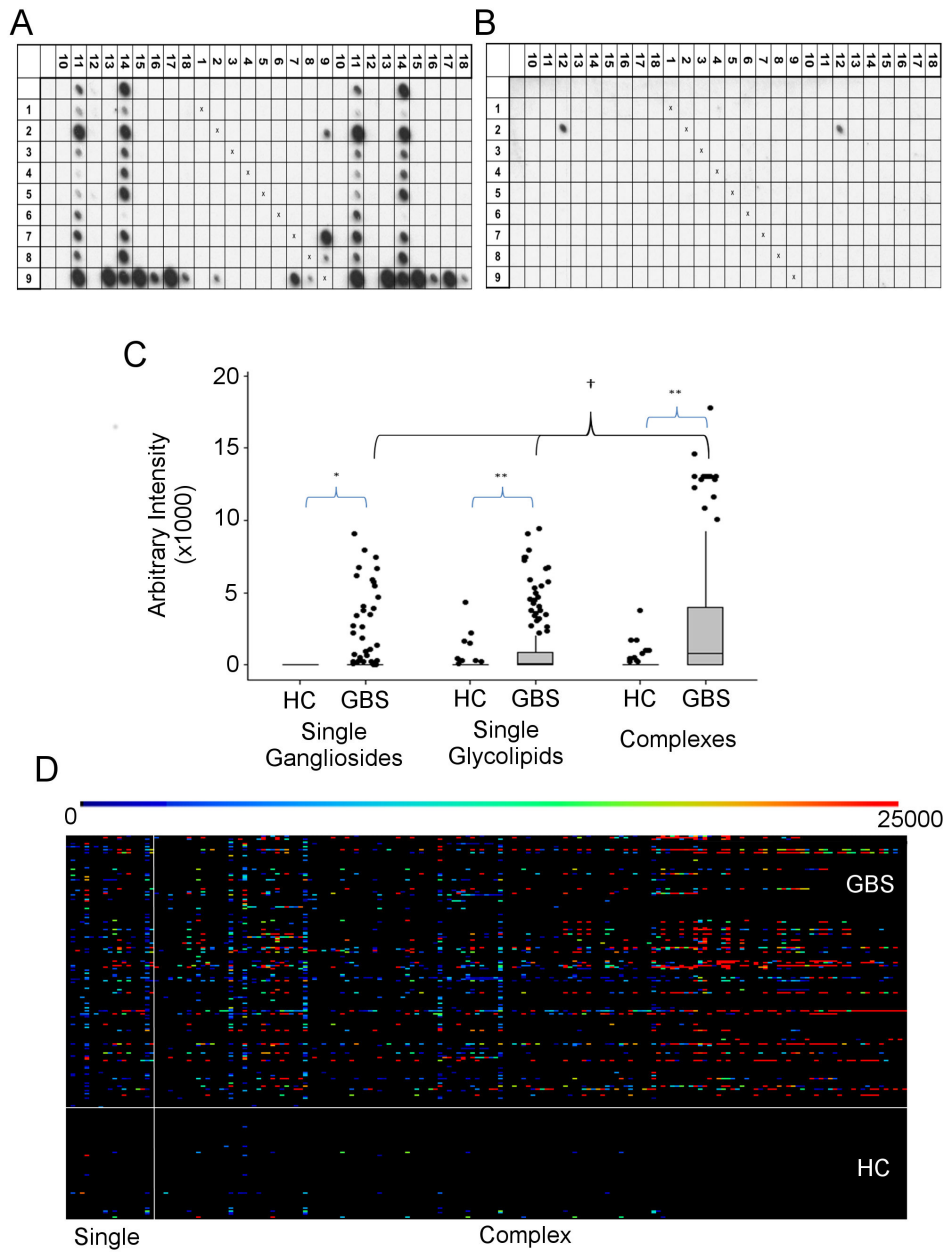
Diverse patterns of glycolipid binding in GBS sera. (**A**,**B**) Representative 28x10 arrays. The antigen spotted at each grid location is revealed by combining the row and column headings given in the key below. Thus, locations (9,7) and (7,9) contain GA1:GM3 complexes. The line of ‘x’ across the centre of the membrane represents the negative control spots (methanol only spotted). The left most column and upper most row contain single antigens only. (**A**) An array probed with GBS serum showing both complex attenuated and enhanced binding patterns. GD1b binding (14) is completely attenuated in complex with GD2 (location 14,6). In contrast, despite no demonstrable binding to GA1, GM3 or GD1a in isolation, an intense spot is seen with both GA1:GM3 (location 9,7) and GA1:GD1a (location 9,13) complexes. This is complex enhanced/complex dependent binding. (**B**) Highly specific complex-dependent GBS sera. In this example, only the duplicate spots of GM1:phosphatidylserine complex (location 2,12) are bound. (**C**) Boxplot of the distribution of maximum binding intensity for GBS versus healthy control (HC) sera assessed for single gangliosides, single glycolipids, and glycolipid complexes. Maximum binding intensity is significantly higher for GBS sera versus healthy control (HC) sera for each antigen group (*p<0.05, **p<0.0001). GBS sera maximum binding intensity for complexes is significantly higher than for either single glycolipids or gangliosides (†=p<0.001), but HC sera maximum binding intensity is insignificantly different between the antigen groups (not marked). (all Kruskal-Wallis/Mann-Whitney). (**D**) Overview heatmap of raw, unclustered binding intensities for GBS (above horizontal white line) and HC (below horizontal white line) sera. Antigens to the right of the vertical white line are glycolipid complexes. The intensity of binding is given by the bar above the heatmap, ranging from black (negative) to red (most intense). A more detailed, zoomable version of this heatmap, allowing resolution of results for each antigen and each serum, is available online (Figure S1). Key: (1)Sphingomyelin, (2)Phosphatidylserine, (3)Globoside, (4)Cereamide trihexosides (CTH), (5) Sulfated glucuronyl paragloboside (SGPG), (6)GM2, (7)GM3, (8)GD2, (9)Asialo-GM1, (10)Galactocerebroside (GalC), (11)LM1, (12)GM1, (13)GD1a, (14)GD1b, (15)GD3, (16)GQ1b, (17)GT1b, (18)Sulfatide.

Following initial screening using 11x11 and 28x10 arrays, IgG antibodies in the sera of 113 GBS patients (62.4%) versus 11 controls (14.9%) bound to one or more antigens (p<0.001, Chi-squared). GBS-associated sera displayed many different binding patterns towards glycolipids and complexes (Figure **1A,B,D**). No single antigen binding pattern was dominant. Overall, GBS sera bound a wider range of antigens at higher intensity compared to healthy controls (Figure **1C,D**). Reactivity towards 176 different single antigens and complexes was seen with sera from GBS patients, whereas healthy control sera only bound 31 of the 181 different antigens (p<0.0001, Binomial). Figure **S1** provides a detailed, zoomable version of this heatmap showing binding patterns for each individual antigen and serum. GBS patients testing positive were an average of 2.5 years older than those testing negative, whereas healthy controls testing positive were an average of 2.2 years younger than those testing negative. However, neither of these differences reached statistical significance (p=0.375 and p=0.589, independent samples T-test). 

#### Low frequency and high specificity antibodies

78 antigens were bound by IgG antibodies found in less than 10 of the GBS sera and 22 antigens were recognised by only 1 or 2 sera. None of these 78 antigens were bound by IgG antibodies in any of the 74 healthy control sera. Other GBS sera displayed very restricted specificity - 13 sera showed binding against 1 or 2 antigens only (Figure **1B**, further examples Figure **S2A**). Additional GBS sera displayed more promiscuous binding to multiple different antigens (Figure **S2B**) 

### Glycolipid complexes

**Figure 2:**
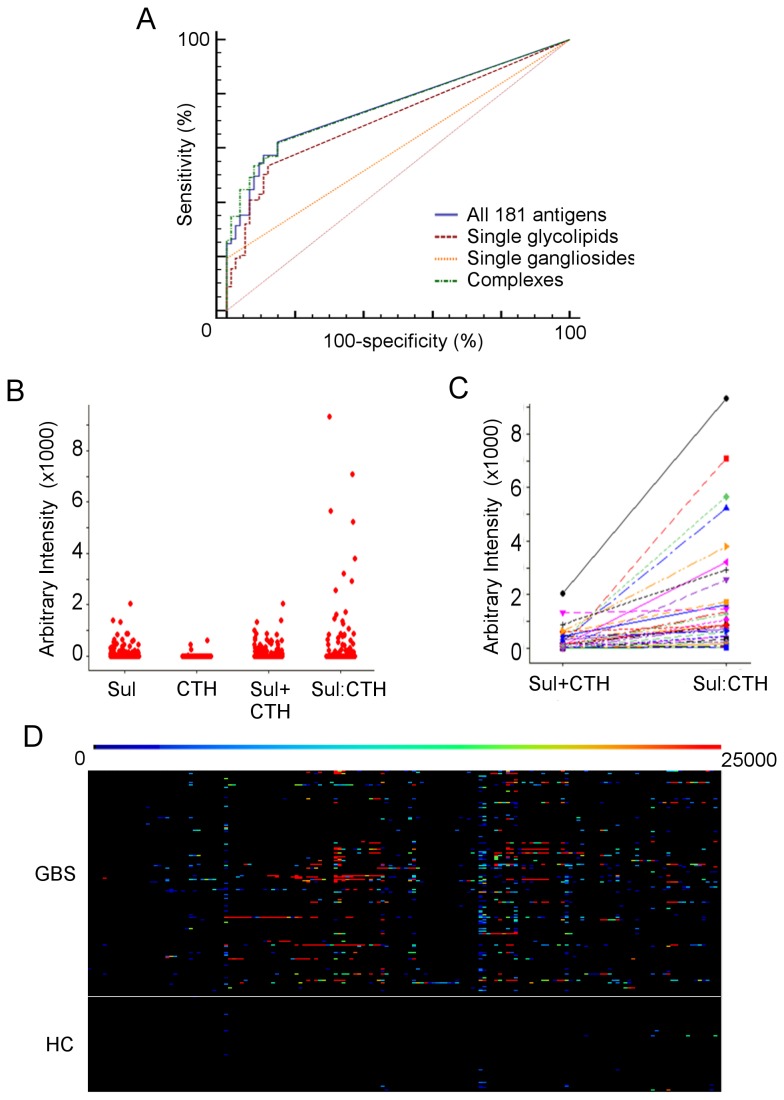
Complex enhanced binding. (**A**) Receiver operator curves for single ganglioside antigens, single glycolipids, glycolipid complexes, and for all 181 antigens. Complex antigens (dashed green line) afford the best trade-off between sensitivity and specificity. (**B**) Individual value plots for GBS sera binding intensity to single sulfatide (sul), single CTH, the sum of these single intensities (sulfatide+CTH), and CTH:sulfatide complexes. (**C**) Comparison between the sum of single sulfatide and CTH binding intensities (sul+CTH) and binding intensity to CTH:sulfatide complexes in individual sera. Each line links the respective values in a single serum. Lines sloping upwards from left to right indicate complex enhanced binding with a single serum. (**D**) Overview heatmap of corrected complex binding intensities. The intensity value for each complex has been corrected by subtracting the binding intensities of each component glycolipid. Any residual intensity indicates complex enhanced binding. As before, GBS sera lie above and healthy control (HC) sera below the horizontal white line. The same colour scale as Figure 1 has been used. A more detailed, zoomable version of this heatmap, allowing resolution of results for each complex antigen and each serum, is available online (Figure S3).

Overall, complexes are bound at a significantly higher median intensity compared to either single gangliosides or glycolipids (Figure **1C**, p<0.001, Kruskal-Wallis/Mann-Witney). Furthermore, if binding to the 11 single gangliosides alone were assessed by glycoarray, the positivity rate would be 80% for AMAN cases, but only 11.8% for those with demyelinating electrophysiology. In contrast, glycolipid complexes are bound by IgG antibodies in 61.2% of sera from demyelinating cases. Overall, the use of glycolipid complex binding intensity values alone provides the best trade-off between sensitivity and specificity for discriminating GBS from healthy control sera, significantly outperforming single ganglioside and/or single glycolipid results, as assessed by receiver operator curve (ROC) analysis (p<0.0001 and p<0.001 respectively) (Figure **2A**). Only the assessment of glycolipid complexes (AUC 0.758) or the inclusion of all 181 antigens (AUC 0.756) resulted in a test with an area under the curve (AUC) value in excess of 0.75, the accepted cut-off for a clinically useful test [29]. The performance of these two antigen groups was insignificantly different (p=0.33, ROC analysis). 

In many cases the complex binding intensities produced by GBS sera were greatly in excess of those seen towards the component glycolipids of the complex assessed individually – a phenomenon referred to as complex enhanced binding [18] (example in Figure **2B,C**). This pattern was much more frequent (59.7% versus 13.5%, p<0.0001, Chi-squared) and pronounced (median complex enhancement 5300 versus 0 arbitrary units, p<0.0001, Mann-Whitney) with GBS versus healthy control sera, as shown by the second heatmap (Figure **2D**). This heatmap displays binding intensities for glycolipid complexes following subtraction of single glycolipid binding intensities (high resolution, zoomable version showing the pattern of enhancement for each complex antigen and serum available as Figure **S3**). Complex enhancement was exclusively seen with GBS sera for 134 of the 161 complexes tested.

Binding to a glycolipid complex without any detectable binding whatsoever to the component glycolipids presented individually has been termed ‘complex dependent’. Overall, 79 (43.6%) GBS sera showed this pattern, compared to only 3 (4.1%) of the healthy control sera (p<0.0001, Fisher’s Exact Test). Examples were seen involving all of the glycolipids assayed (Figure **S2C**). 

### Individual antigens with significantly increased binding intensities

Logarithmic transformation of the intensity values, and analysis by mixed effects model ANOVA with Bonferroni correction, revealed 9 antigens with significantly increased binding intensities in GBS as compared with healthy controls. Seven of these were glycolipid complexes. Using the less stringent Benjamini and Hochberg step-up correction for multiple comparisons, a further 31 significant antigens (28 complexes) are returned (Table **1**). The same technique was used to identify specific antibody-antigen/intensity associations for disease subtype (by electrophysiological criteria), disease severity, identified preceding infection, cranial nerve involvement, and the requirement for mechanical ventilation (Table **2**). 

**Table 2:** Clinical features significantly associated with specific antigens.

**Clinical Feature**	**Significantly associated antigens**
Axonal Electrophysiology	CTH :GM1, GD2:GM1, Globoside:GM1, GalC:GM1, GA1:GM1, GM1, GM1:GD1b, SM:GM1, GM3:GM1, GM1:GD3, SGPG:GM1, GM2:GM1, PS:GM1, Sulfatide:GM1, GA1:GalC, GM1:GQ1b, GA1, GA1:Sulfatide
Greater Disease Severity	CTH :GA1*, Globoside:GA1*, PS:GalC, GA1:GalC, Globoside:GD3, GD2:GD3, GA1, GD3:GT1b, GalC:GQ1b
Preceding *Campylobacter jejuni* infection	GD1a:GT1b, GD3:GD1a, Sulfatide:GD1a, GalC:GD1a, GM2:GD1a, GD1a, GD1a:GQ1b, GD2:GD1a, CTH:GD1a
No cranial nerve deficit	Sulfatide:GM1, Sulfatide:GT1a

Following Bonferroni correction, 18 antigens (including GM1 and 14 GM1 containing complexes) were significantly positively associated with the AMAN variant of GBS, as compared with neurophysiologically confirmed AIDP.

Nine antigens were statistically significantly positively associated with severe disease (GBS disability score >3), but only at the point of randomisation (day 0). At other assessed time points, this association is not seen. Antigens marked with an asterisk were also significantly independently associated with the requirement for mechanical ventilation.

Antigen-antibody associations with preceding *Campylobacter jejuni* infection comprise solely of GD1a and GD1a series complexes.

(SM – sphingomyelin, PS – phosphatidylserine, GalC – galactocerebroside, GA1 – asialo-GM1)

### Antibody binding patterns in other neurological disease control sera

The 40 antigens identified by intensity analysis as significantly associated with GBS were then subjected to testing with the 87 OND controls, alongside a randomly selected subset of 20 anti-GSC antibody positive GBS sera. Comparing the results of the retested GBS sera with those from the original 28x10 assay, there were no significant differences in positivity rates (using Fisher’s Exact test) or average signal intensities (by Mann-Whitney) for 35 of the 40 antigens. All five of the discrepant antigens (sulfatide:GT1a, LM1:GT1a, GM1:GT1a, GalC:GT1a, and SGPG:GalC) were excluded from further analysis. 

**Figure 3:**
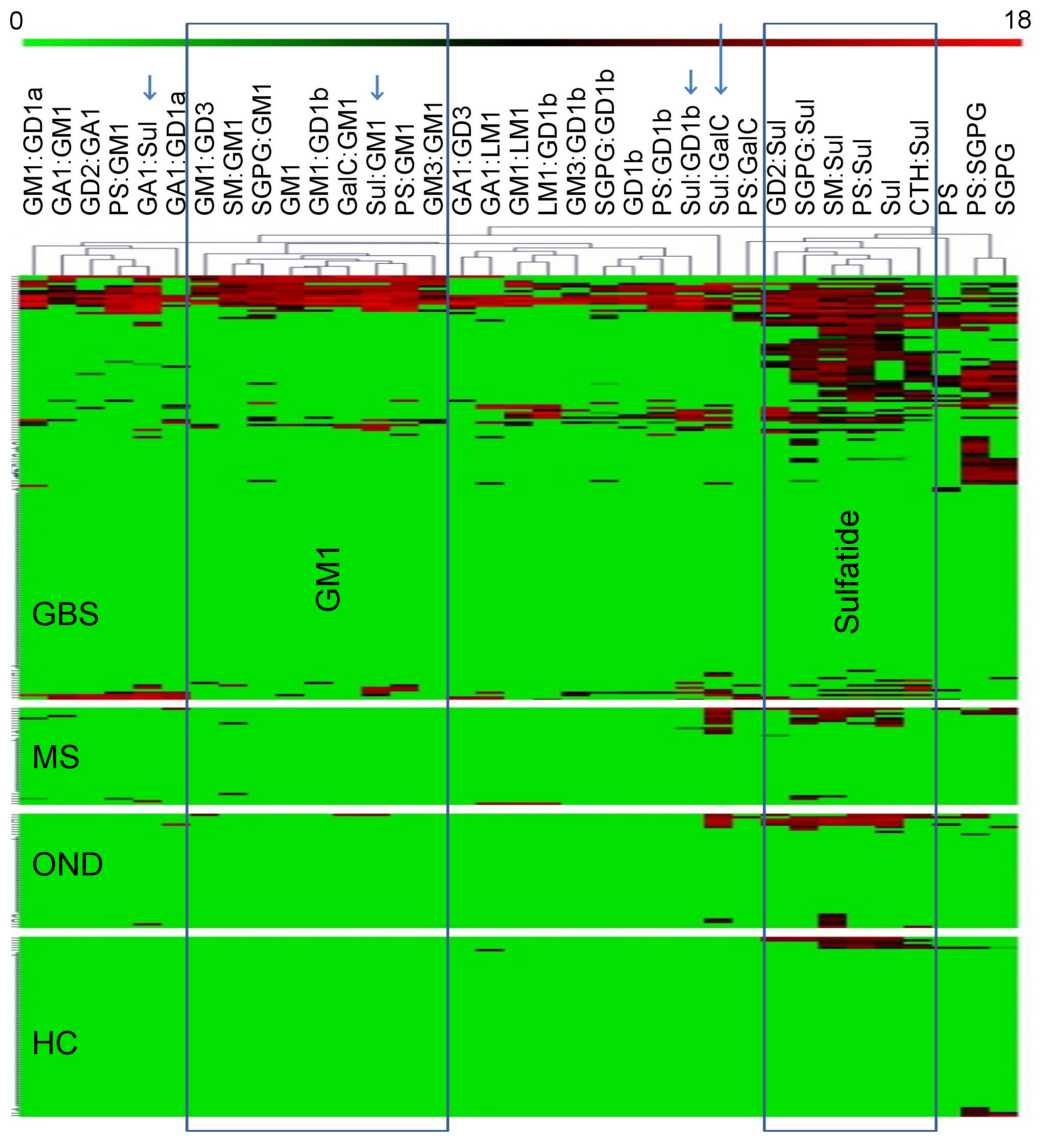
Heatmap of glycolipid complex binding in GBS and control sera. Clustered heatmap for the 35 antigens significantly associated with GBS (arranged on the x-axis), comparing the 181 GBS sera and 161 control sera (on the y-axis). Each row represents a single serum. The log transformed arbitrary binding intensity for each antigen is represented by the colour scale, from red (log transformed arbitrary intensity of 18, most intense) to the green background (log transformed arbitrary intensity of 0, no binding), as shown at the top of the figure. Each heatmap panel represents a separate group, as labelled. (GBS, Guillain-Barré syndrome; MS, Multiple Sclerosis; ONND, Other Non-Inflammatory Neurological Diseases; HC, Healthy Controls). Blue boxes delineate clusters of GM1 and sulfatide-complex antigens. Arrows mark sulfatide complexes which cluster separately.

**Figure 4:**
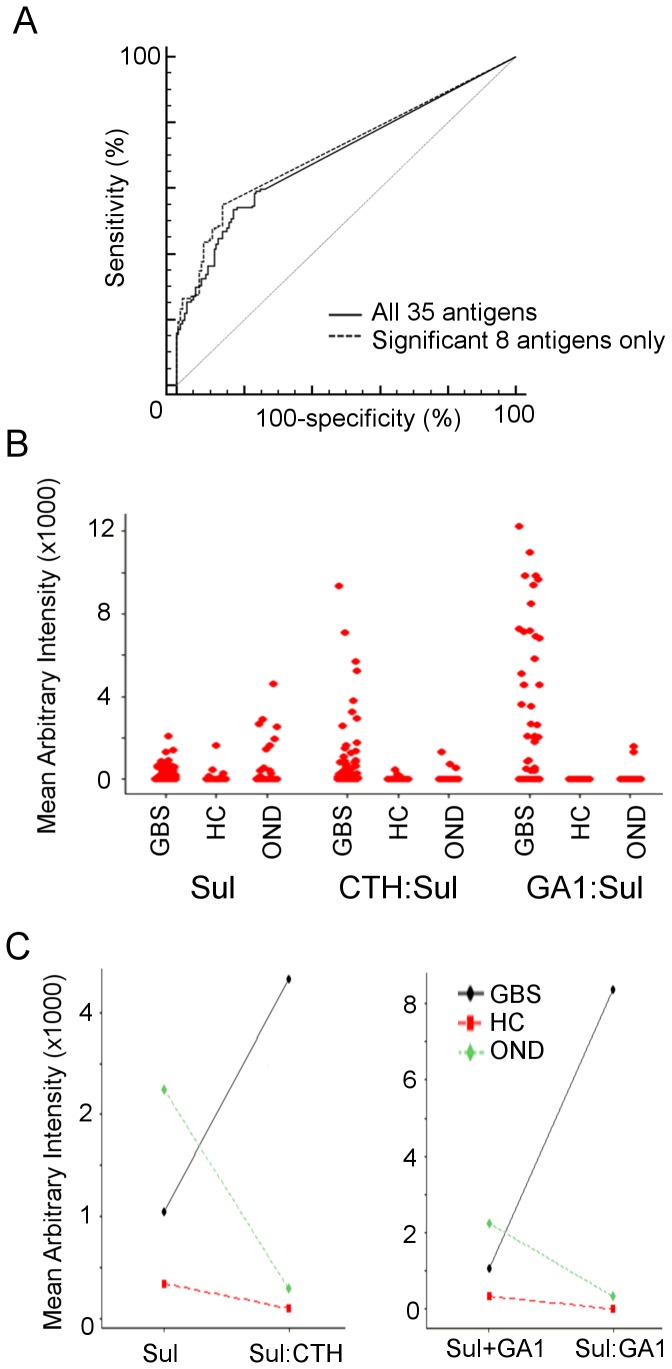
Differing patterns of sulfatide complex enhanced binding in disease versus control sera. (**A**) The receiver operator curve for the 8 antigen intensities significantly associated with GBS versus OND controls is insignificantly different to that produced by including all 35 antigens. (**B**) Individual value plots of binding intensity for GBS, healthy control (HC), and other neurological disease (OND) sera to sulfatide, CTH:sulfatifde complex, and GA1:sulfatide complex. (**C**) Mean change in complex compared to summed single antigen binding intensity for CTH:sulfatide and GA1:sulfatide. On average, GBS sera show complex enhancement, whereas HC and OND sera show complex attenuation.

Hierarchical clustering using a Pearson’s Correlation metric was used to uncover patterns of binding in GBS associated sera, which was then compared to that seen in controls. This revealed two major GBS binding clusters – one containing sulfatide complexes and the other GM1 complexes (boxed in Figure **3**). Positivity in control cases mainly mapped to the sulfatide cluster. Four sulfatide complexes cluster separately, three of these (GM1:sulfatide, GD1b:sulfatide and GA1:sulfatide, small arrows) are infrequently represented in controls, Sulfatide:GalC (large arrow) binding is present in OND controls at a frequency similar to other antigens in the sulfatide cluster. Eight antigens displayed a significantly increased binding intensity with GBS as compared to OND control sera (Table **3**), including GM1, 3 GM1 complexes, and 4 sulfatide complexes. The ROC/assay performance of this subset of 8 antigens is insignificantly different to that of all 35 antigens together (p=0.32, Figure **4A**).

**Table 3:** Antigens with significantly increased binding intensities for GBS versus OND sera.

**Antigen**	**Corrected (Bonferroni) (p value)**	**Sensitivity **	**Specificity **	**AUC**
Phosphatidylserine:Sulfatide^*^	0.00018	37%	90.7%	0.631
CTH:Sulfatide^*^	0.00092	27.1%	96.3%	0.617
Phosphatidylserine:GM1	0.0024	14.4%	100%	0.575
Phosphatidylserine:SGPG*	0.0050	28.7%	95%	0.615
SGPG:GM1	0.0056	13.3%	100%	0.566
Sulfatide:GM1	0.0077	13.8%	99.4%	0.566
GM1	0.024	11%	100%	0.555
GA1:Sulfatide*	0.034	17.7%	98.8%	0.583
(Above 8 Antigens Overall)		(55.2%)	(85.7%)	(0.716)

The eight antigens above had significantly increased GBS sera binding intensities versus OND controls, following Bonferroni correction, and all also had significantly increased positivity rates (corrected p value <0.05). Antigens marked with an asterisk also showed significantly increased binding intensities versus normal controls following Bonferroni correction. All of the antigens listed also had significantly increased binding intensities versus healthy controls following the less stringent step-up correction. The sensitivity and specificity values given are based on a cut value of zero (i.e. a binding intensity value greater than zero is considered positive).

(GA1 – asialo-GM1, CTH – trihexosylceramide (ceramide trihexosides), SGPG - sulfated glucuronyl paragloboside, AUC = area under curve, from Receiver Operator Curve analysis)

### Sulfatide and sulfatide series complex antibodies

Sulfatide-complex antibodies form the largest group of antibodies found to be statistically associated with GBS. Sulfatide as a single glycolipid is bound at relatively low intensity by IgG in GBS sera, there is some overlap with healthy control binding intensity, and no significant difference in binding as compared to OND control sera. Once again however, disease binding intensity and separation from control cases is substantially improved with certain sulfatide complexes, such asialo-GM1:sulfatide and CTH:sulfatide (Figure **4B**). Furthermore, there is a differential relative response to single sulfatide and sulfatide-complex antigens between GBS and control sera. Overall, GBS sera show a more frequent and more pronounced complex-enhanced response to these sulfatide-containing complexes than do OND or healthy control sera (Kruskal-Wallis test for *degree* of complex enhancement, CTH:sul p=0.025, GA1:sul p=0.037, both p<0.001 when corrected for ties, Chi-squared comparison of *frequency* of complex enhancement, p<0.0001 for both antigens) (Figure **4C**). 

### GM1 and GM1 series complex antibodies

**Figure 5:**
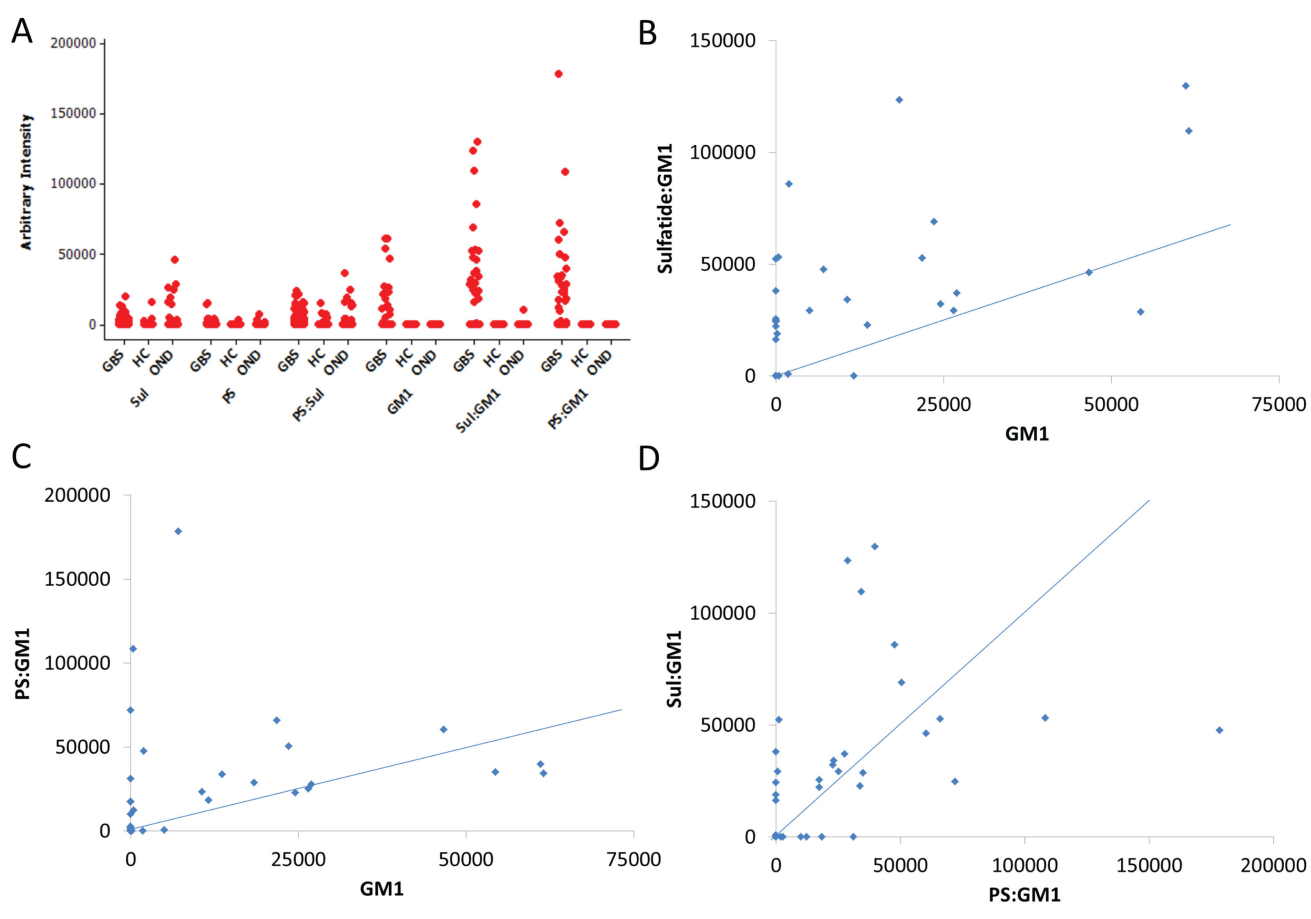
GM1 complex antigens. (**A**) Individual value plots for selected antigens. (**B**) Association between sulfatide:GM1 and GM1 binding intensities in individual sera. Points on the y-axis are GM1 negative, sulfatide:GM1 positive. Sera close to the diagonal blue line (joining points of identical binding intensity for both antigens) bind both GM1 and sulfatide:GM1 complex with similar intensity. (**C**) Association between phosphatidylserine:GM1 and GM1 binding intensities in individual sera. Points on the y-axis are GM1 negative, PS:GM1 positive. Sera on the x-axis are GM1 positive, PS:GM1 negative. (**D**) Association between phosphatidylserine:GM1 and sulfatide:GM1 binding intensities in individual sera.

In contrast to sulfatide, binding to single GM1 is significantly increased in GBS compared to OND control sera (Table **3**). Even so, GM1 complexes have greater sensitivity and increased binding intensity over GM1 alone, without a significant loss of specificity (Figure **5A**). Analysis of relative binding intensities in single patients reveals that both PS:GM1 and sulfatide :GM1 complexes are often bound by sera from patients entirely negative for GM1 reactivity (Figure **5B,C**). The reverse also occurs, but is less often seen. Furthermore, investigating the association between PS:GM1 and sulfatide:GM1 binding intensity in individual sera uncovers at least three different populations of GM1-complex antibodies – those which preferentially (or indeed exclusively) bind one complex, and those which bind both relatively equally (Figure **5D**).

### Comparison with ELISA

Just 39 of the 180 (21.7%) GBS sera had been positive when tested contemporaneously using a conventional GM1, GD1a, and GQ1b ELISA [25]. For these three ganglioside antigens alone, the array detected binding in only 27 cases, and was negative for 15 of the sera which had been positive on ELISA. Only 9 of 85 cases (10.6%) with demyelinating pattern electrophysiology were positive by ELISA (versus 62.4% for binding to all antigens on the array), compared to 4 of the 5 AMAN patients (80%, identical to the array). The remainder of ELISA positive cases had either equivocal (24 patients) or unavailable (2 patients) electrophysiology results. Only one GBS serum which was positive by ELISA was entirely negative by glycoarray. 

## Discussion

The combinatorial glycoarray technique has substantially increased the number of glycolipid antibody specificities significantly associated with GBS. Glycolipid complexes are important both for the overall detection rate and for revealing differences in the fine antibody specificities of disease versus control sera. Although antibodies against single gangliosides are frequently found in axonal disease subtypes, glycolipid-complex antigens are more important for increasing the positivity rate in cases labelled as AIDP or ‘equivocal’ by the electrophysiological methods in use at the time of the clinical study. 

Rather than identifying a single specificity, however, the antibody response in GBS is shown to be diverse and includes extensive complex enhanced binding. Whether this reflects some aspect of the underlying dysimmune process in GBS is unclear. It is interesting to speculate that a change in antibody specificity from a single to complex response is a key part of the immune-pathological process. Even without any assertion of pathogenesis, however, it is apparent that there is a generalised upregulation in the glycolipid complex IgG response in GBS. This does not appear simply to be a function of increasing age. In contrast to traditional models of humorally mediated pathology invoking a single antigen target for each disease, the response in GBS has a highly variable fine specificity towards many different neuronal and myelin glycolipid complexes, and there is evidence from this study that this correlates with the variable clinical presentation. That an additional, dominant, antibody specificity has thus far escaped detection is perhaps less likely.

Until recently, Western GBS has been considered to be predominantly AIDP. New observations dependent upon serial electrophysiological studies now suggest that a proportion of cases labelled as AIDP (or equivocal) by a single study may instead have axonal pathology [30]. Serial nerve conduction studies are not routine, and the true proportions of demyelinating and axonal subtypes in different geographical populations is therefore unknown. Nevertheless, even amongst the small number of axonal cases in our series, GM1 and GM1 complexes were bound at significantly greater intensity, and single gangliosides at significantly increased frequency, as compared to cases with a demyelinating and/or equivocal electrophysiological categorisation. This is consistent with the previously reported clinical-serological correlations for the axonal disease subtype [31].This GBS cohort was geographically defined and is representative of the local mix of GBS subtypes. As such, overall positivity rates will be reflective of those in GBS cases in this region, regardless of the true proportion of pathologically axonal or demyelinating cases. Care needs to be taken in correlating patterns of antibody reactivity with *pathological* subtypes when only a single neurophysiological study has been performed. Likewise, the discrepancy between ELISA and glycoarray results highlights the way in which different surfaces can alter antibody binding patterns to glycolipids, possibly through differences in antigen conformation and accessibility, raising the possibility that antibody-glycolipid interactions in live membranes may be different still. 

The pathogenic potential of complex antibodies remains speculative and incompletely proven. *Ex vivo* studies on murine neuromuscular tissue have revealed that some GM1:GD1a complex antibodies from AMAN cases bind, activate complement, and pathologically effect neurophysiological function of axon terminals within the neuromuscular junction [16]. Whether the larger population of anti-glycolipid complex antibodies described herein have such an effect, or alternatively result in complement mediated damage at sites within the node of Ranvier or to compact myelin [32], remains to be seen. Even in diseases traditionally thought to be ‘non-inflammatory’, glycolipid complex antibodies are detected at an increased frequency compared to healthy controls, albeit at lower binding intensities than in GBS. Binding to single sulfatide is widely seen in many disease states, perhaps reflecting non-specific antibody interactions with the sulfated headgroup, whereas antibodies binding certain sulfatide complexes are specifically associated with GBS. The importance of these complex antibodies for GBS and other diseases is unclear. They may be epiphenomena arising as a by-product of antigen release in tissue damage or represent a non-specific up-regulation of humoral immunity in post-infectious states. In this GBS cohort, only single serum samples collected early in the disease course were available for analysis. Therefore, from this study the temporal relationship between the detected antibodies and the disease cannot be firmly established. Larger cohort studies collecting longitudinal data are required to address this, and are on-going [33]. Distinguishing the nature and pathological effects of the disease relevant antibody component from polyclonal background in sera is also problematic, and will require the development of anti-complex monoclonal antibodies.

Very limited information is available on the existence or distribution of glycolipid complexes *in vivo*, although when identified they are known to have profound effects on antibody binding [15]. It is likely that a range of complexes exist in different anatomical locations, but this remains to be demonstrated. Their presence could explain many unresolved enigmas in GBS. 

The technology described here can readily be applied to other areas of humoral immunity and autoimmunity in which lipids and glycolipids are targets, and enables rapid screening against a large range of glycolipid antigens. A general re-examination of research in the area of anti-lipid/glycolipid antibodies is required, taking into account the additional considerations introduced by complexes. Such approaches may allow the detection of other, previously elusive, autoantibodies. 

## Supporting Information

Figure S1 **High resolution unsorted heatmap of binding intensities.** .GBS patients (above horizontal white line) and healthy controls (below horizontal white line) are compared. Antigens are listed on the x-axis such that each column of the heatmap represents the range of binding seen for that antigen. Each row represents the range and intensity of antigen binding seen in an individual patient/serum. The intensity of binding is given by the bar above the heatmap, ranging from black (negative) to red (most intense).
(TIF)

Figure S2 **Glycolipid complex binding patterns of GBS sera.** Both highly specific (A) and promiscuous (B) binding were seen with different GBS patient sera. Many sera showed absolute complex dependent binding. In these cases, sera bound a heterodimeric 1:1 complex without any detectable binding to either component glycolipid presented individually. This phenomenon was observed 828 times (as some sera demonstrated multiple examples). Most often, phosphatidylserine (pulled out slice) was the partnering glycolipid in this situation, but examples were seen involving all of the glycolipids assayed (C).
(DOCX)

Figure S3 **Overview heatmap of corrected complex binding intensities.** The intensity value for each complex has been corrected by subtracting the binding intensities of each component glycolipid. Any residual intensity indicates complex enhanced binding. As before, GBS sera lie above and healthy control (HC) sera below the horizontal white line. The same colour scale as Figure S1 has been used.
(TIF)

Table S1 **Diagnoses of patients with other neurological diseases used as controls.** ONND – other non-inflammatory neurological diseases, MS – multiple sclerosis, RR – relapsing remitting, PP – primary progressive, SP – secondary progressive, CIS – clinically isolated syndrome, TM – transverse myelitis, CFS – chronic fatigue syndrome, IIH – idiopathic intracranial hypertension, CVD – cerebrovascular disease, PFO – patent foramen ovale, CVST – cerebral venous sinus thrombosis.
(DOCX)
