# Supplementary figures and images for: Antibodies to Heteromeric Glycolipid Complexes in Guillain-Barré Syndrome
Source: PLoS One. 2013 Dec 16;8(12):e82337. doi: 10.1371/journal.pone.0082337 (PMC3864991; doi:10.1371/journal.pone.0082337)

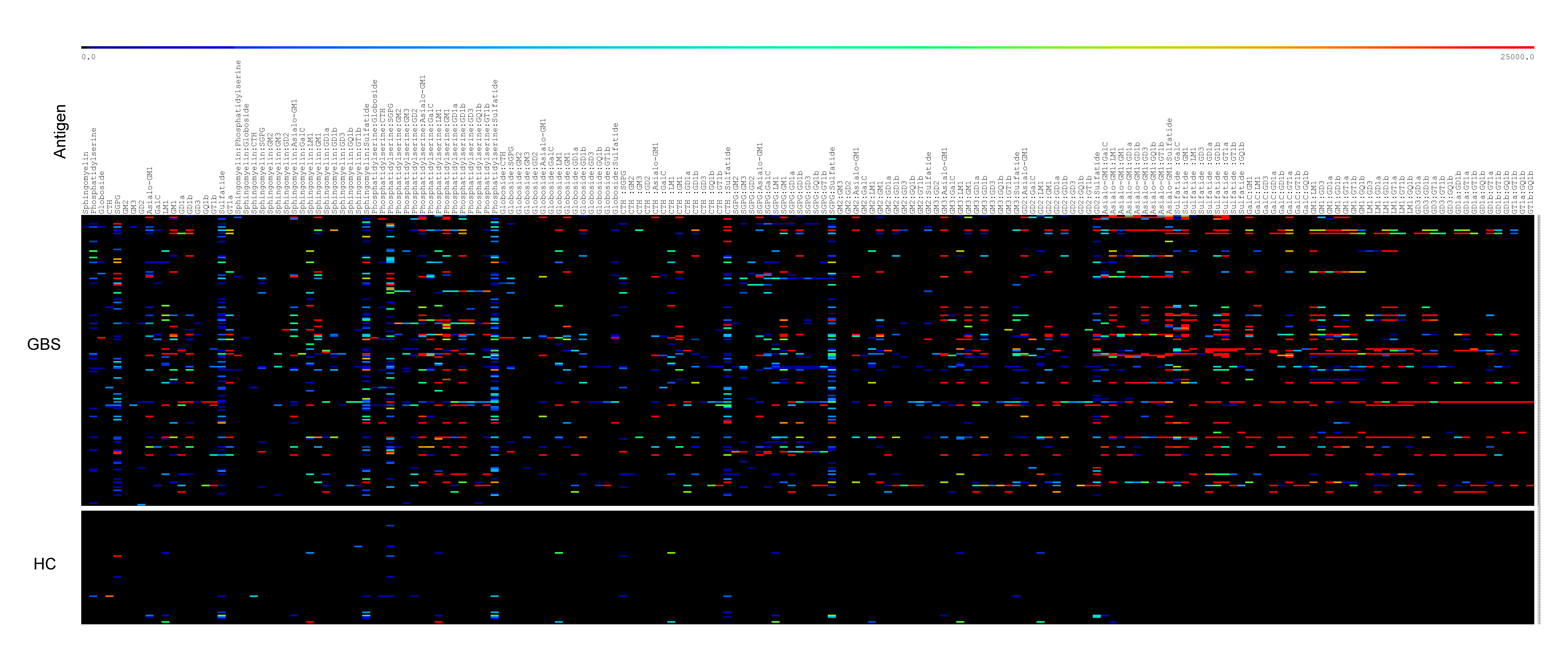

Supplement: Figure S1 — High resolution unsorted heatmap of binding intensities. .GBS patients (above horizontal white line) and healthy controls (below horizontal white line) are compared. Antigens are listed on the x-axis such that each column of the heatmap represents the range of binding seen for that antigen. Each row represents the range and intensity of antigen binding seen in an individual patient/serum. The intensity of binding is given by the bar above the heatmap, ranging from black (negative) to red (most intense). (TIF) [file pone.0082337.s001.tif]

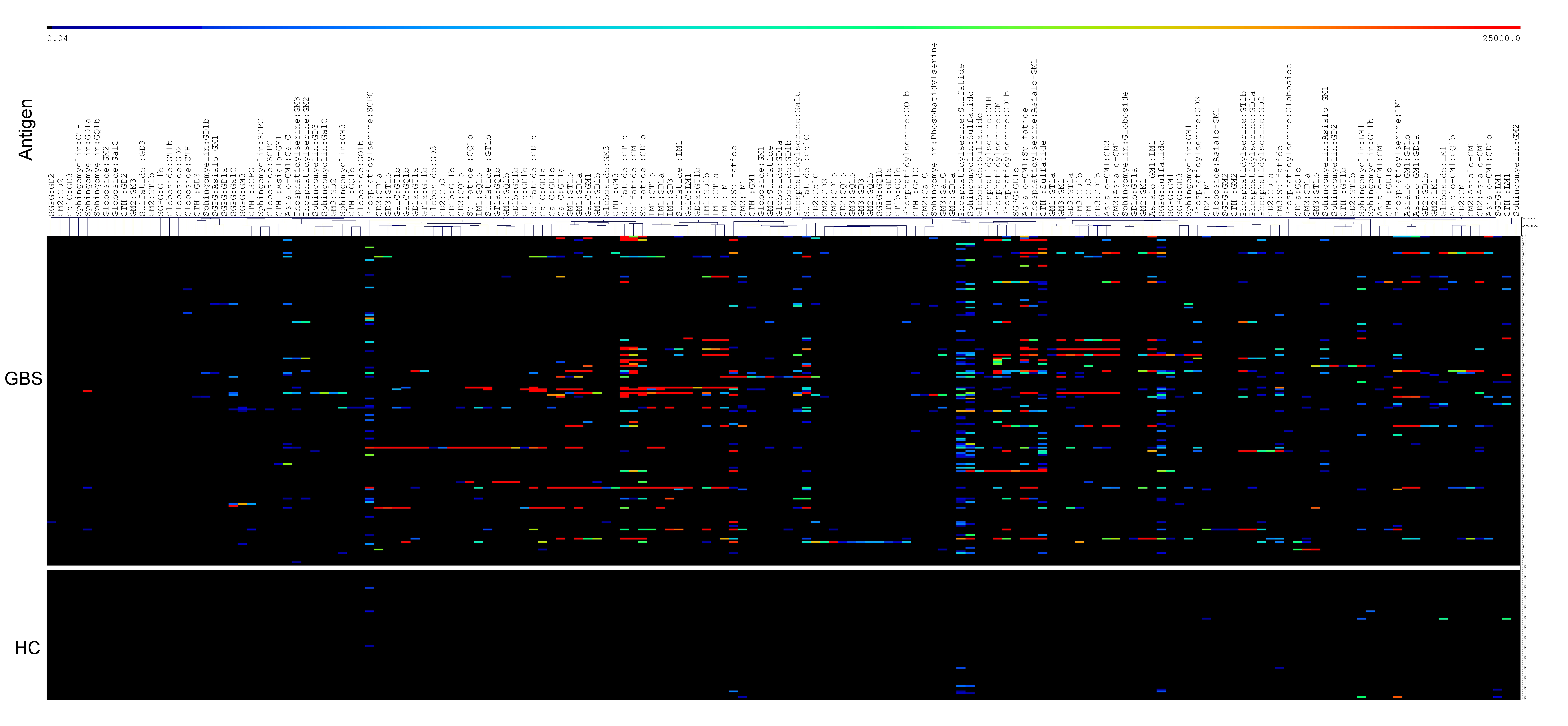

Supplement: Figure S3 — Overview heatmap of corrected complex binding intensities. The intensity value for each complex has been corrected by subtracting the binding intensities of each component glycolipid. Any residual intensity indicates complex enhanced binding. As before, GBS sera lie above and healthy control (HC) sera below the horizontal white line. The same colour scale as Figure S1 has been used. (TIF) [file pone.0082337.s003.tif]
