# Supplementary material for: Antibodies to Heteromeric Glycolipid Complexes in Guillain-Barré Syndrome
Source: PLoS One. 2013 Dec 16;8(12):e82337. doi: 10.1371/journal.pone.0082337 (PMC3864991; doi:10.1371/journal.pone.0082337)
Supplement: Figure S2 — Glycolipid complex binding patterns of GBS sera. Both highly specific (A) and promiscuous (B) binding were seen with different GBS patient sera. Many sera showed absolute complex dependent binding. In these cases, sera bound a heterodimeric 1:1 complex without any detectable binding to either component glycolipid presented individually. This phenomenon was observed 828 times (as some sera demonstrated multiple examples). Most often, phosphatidylserine (pulled out slice) was the partnering glycolipid in this situation, but examples were seen involving all of the glycolipids assayed (C). (DOCX) [file pone.0082337.s002.docx]

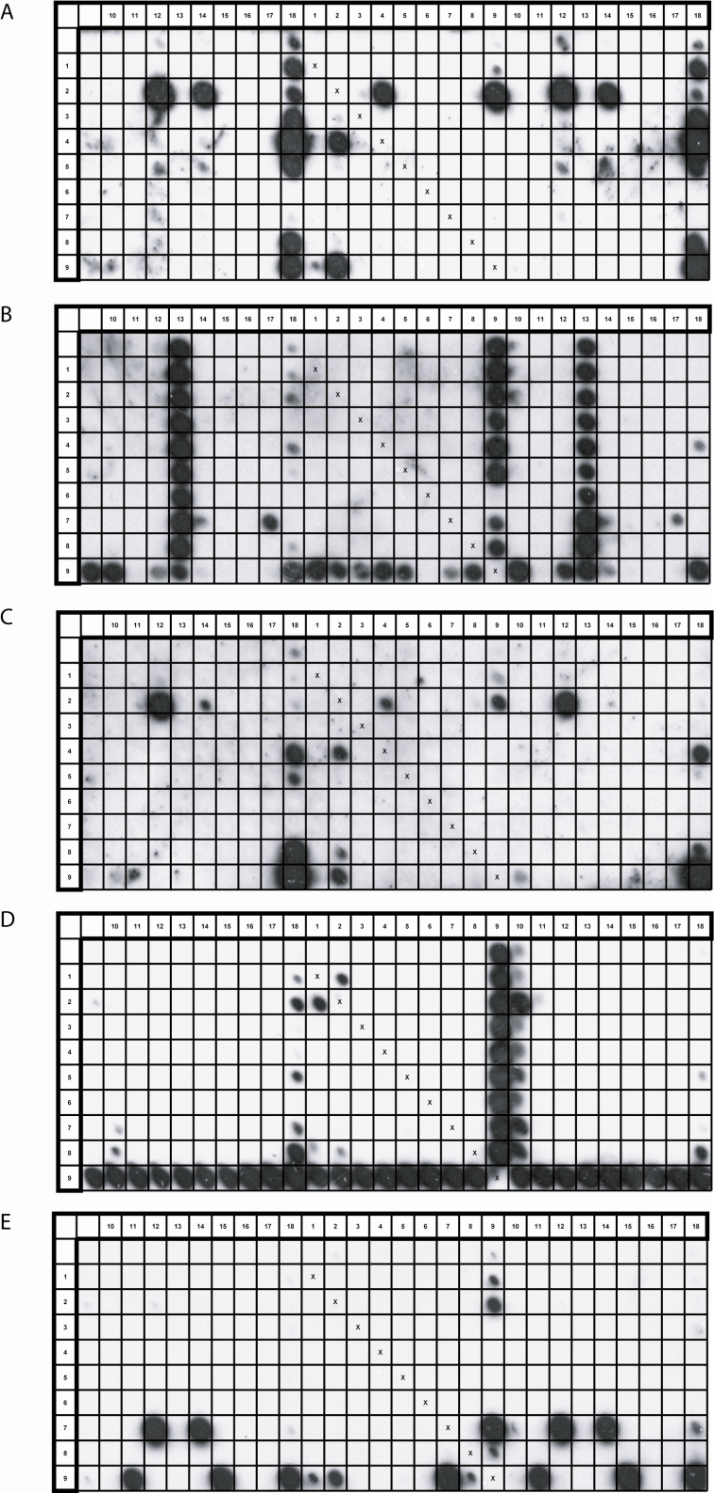

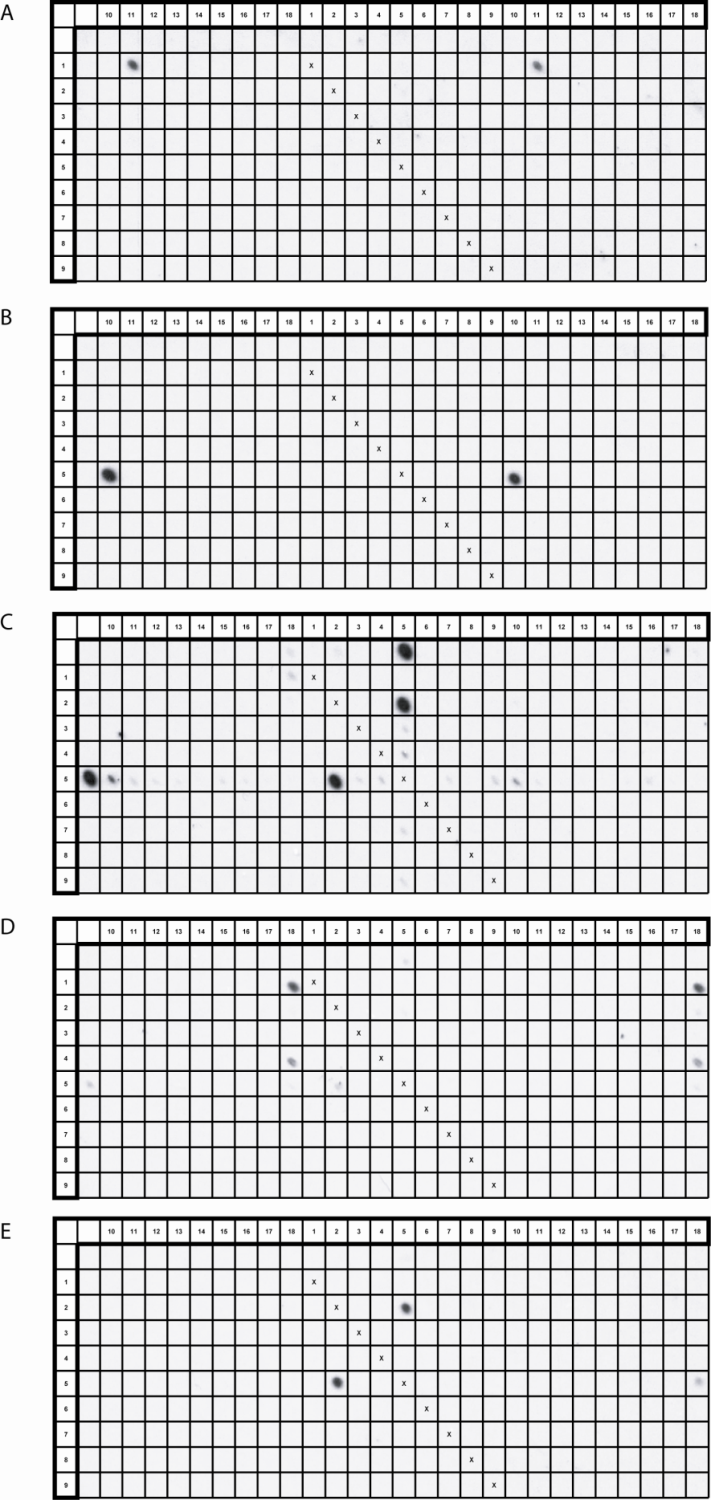


C

B

A

Supplementary figure S2 – Glycolipid complex binding patterns of GBS sera

Both highly specific (**A**) and promiscuous (**B**) binding were seen with different GBS patient sera. Many sera showed absolute complex dependent binding. In these cases, sera bound a heterodimeric 1:1 complex without any detectable binding to either component glycolipid presented individually. This phenomenon was observed 828 times (as some sera demonstrated multiple examples). Most often, phosphatidylserine (pulled out slice) was the partnering glycolipid in this situation, but examples were seen involving all of the glycolipids assayed (**C**).
