# Supplementary material for: Antibodies to Heteromeric Glycolipid Complexes in Guillain-Barré Syndrome
Source: PLoS One. 2013 Dec 16;8(12):e82337. doi: 10.1371/journal.pone.0082337 (PMC3864991; doi:10.1371/journal.pone.0082337)
Supplement: Table S1 — Diagnoses of patients with other neurological diseases used as controls. ONND – other non-inflammatory neurological diseases, MS – multiple sclerosis, RR – relapsing remitting, PP – primary progressive, SP – secondary progressive, CIS – clinically isolated syndrome, TM – transverse myelitis, CFS – chronic fatigue syndrome, IIH – idiopathic intracranial hypertension, CVD – cerebrovascular disease, PFO – patent foramen ovale, CVST – cerebral venous sinus thrombosis. (DOCX) [file pone.0082337.s004.docx]

|  | **ONND** | **MS** |
| --- | --- | --- |
| 1 | Non-organic illness | RRMS |
| 2 | PFO/ CVD | RRMS |
| 3 | CFS | RRMS |
| 4 | Atonic bladder/CFS/non-epileptic episodes | RRMS |
| 5 | Stress | PPMS |
| 6 | CFS | PPMS |
| 7 | Non-organic illness | BS-RRMS |
| 8 | Non-organic illness | SPMS |
| 9 | Vestibulopathy, Migraine | SPMS |
| 10 | IIH, Migraine | CIS |
| 11 | Motor neuron disease | PPMS |
| 12 | IIH | RRMS |
| 13 | IIH | RRMS |
| 14 | Cognitive impairment | RRMS |
| 15 | Viral meningitis | TM/CIS |
| 16 | Migraine | RRMS |
| 17 | Migraine without aura | RRMS |
| 18 | Optic nerve drusen + IIH | RRMS |
| 19 | Glioblastoma | PRMS |
| 20 | Fronto-temporal dementia | PPMS |
| 21 | TB Myelitis | PPMS |
| 22 | Headache- not IIH | RRMS |
| 23 | Non specific headache | RRMS |
| 24 | Primary lateral sclerosis | RRMS |
| 25 | Cerebrovascular disease | RR-TM |
| 26 | IIH | PPMS |
| 27 | Primary lateral sclerosis | RRMS |
| 28 | Primary lateral sclerosis | RRMS |
| 29 | Aseptic meningitis | RRMS |
| 30 | Pseudoseizures | RRMS |
| 31 | Chronic fatigue syndrome | RRMS |
| 32 | Small vessel disease | RRMS |
| 33 | IIH secondary to CVST | RRMS |
| 34 | Small vessed disease | PPMS |
| 35 | L Bell’s Palsy | RRMS |
| 36 | Midbrain infarct | RRMS |
| 37 | CVD | RRMS |
| 38 | Small vessel disease | RRMS |
| 39 | Non-organic illness | RRMS |
| 40 | Myelopathy | RRMS |
| 41 | Idiopathic peripheral neuropathy |  |
| 42 | Small fibre neuropathy, white matter changes- not MS |  |
| 43 | Hereditary motor neuropathy |  |
| 44 | Diabetic neuropathy |  |
| 45 | Chronic axonal polyneuropathy + L ulnar neuropathy |  |
| 46 | Sensory neuropathy |  |
| 47 | Sensory ataxic neuropathy |  |

**Table S1 – Diagnoses of patients with other neurological diseases used as controls**ONND – other non-inflammatory neurological diseases, MS – multiple sclerosis, RR – relapsing remitting, PP – primary progressive, SP – secondary progressive, CIS – clinically isolated syndrome, TM – transverse myelitis, CFS – chronic fatigue syndrome, IIH – idiopathic intracranial hypertension, CVD – cerebrovascular disease, PFO – patent foramen ovale, CVST – cerebral venous sinus thrombosis
